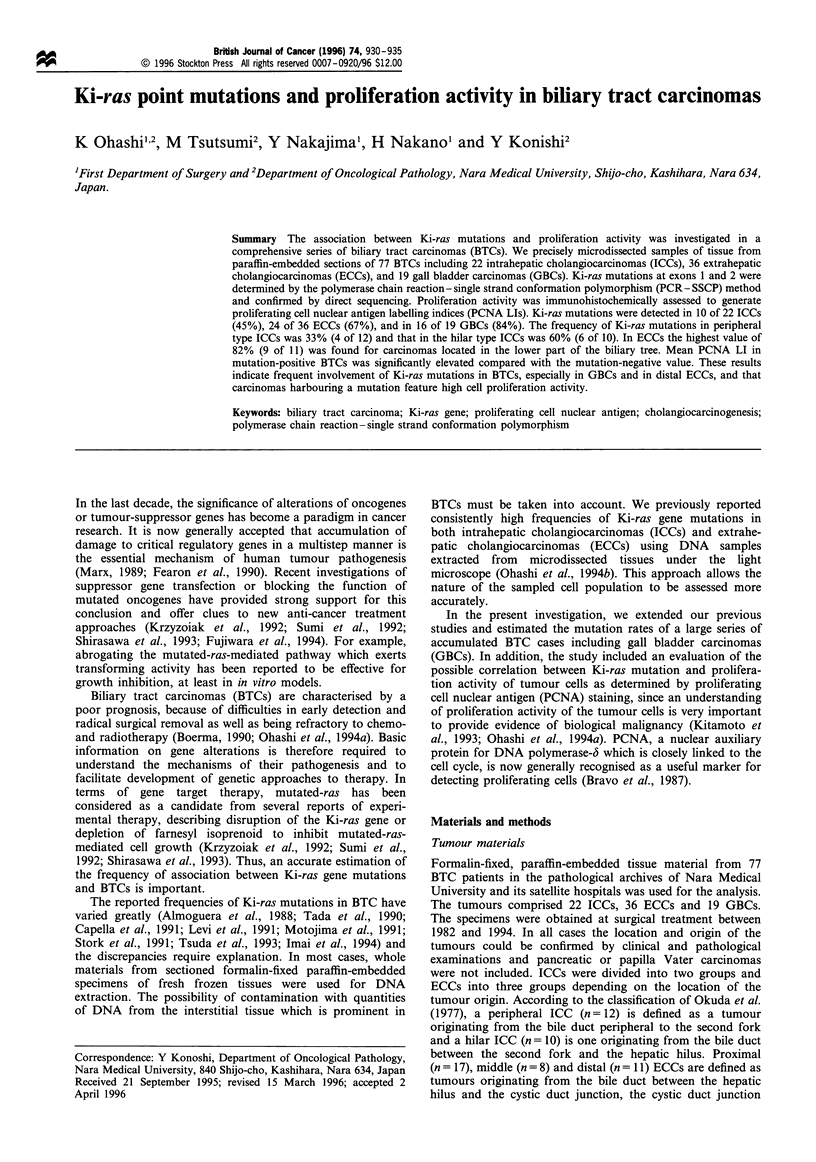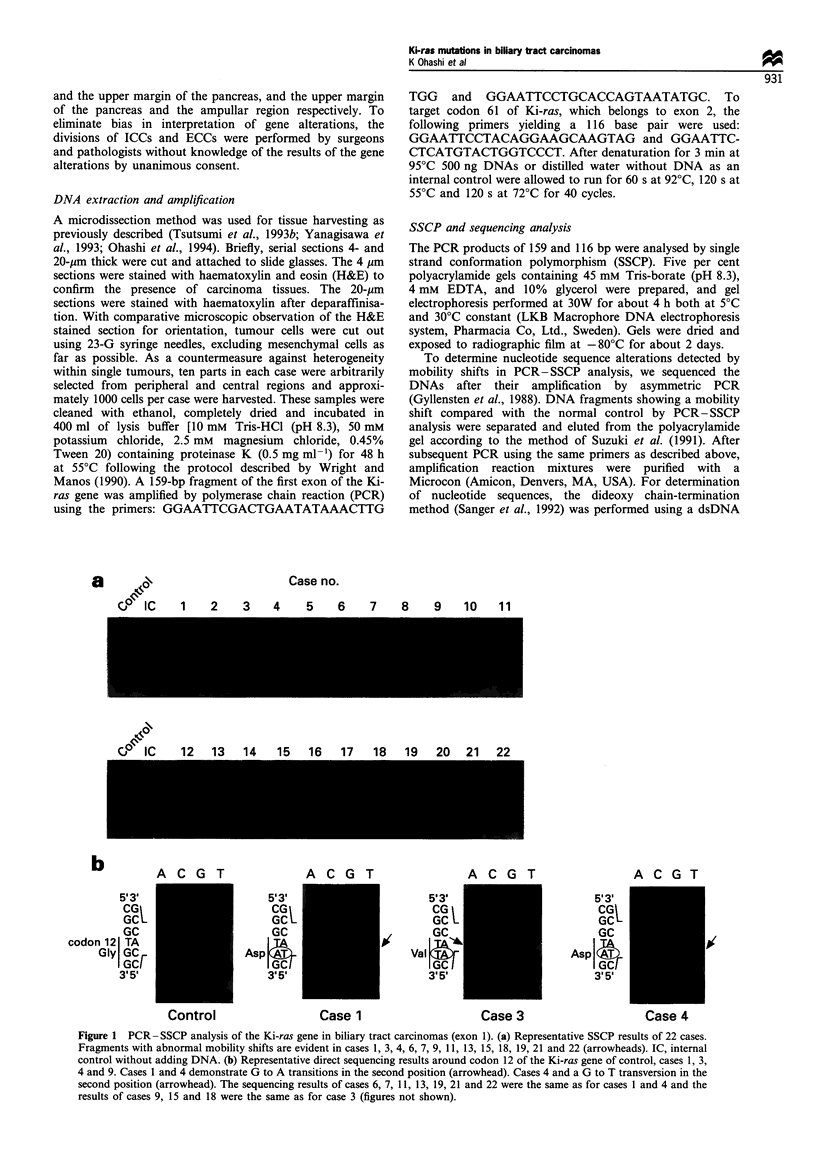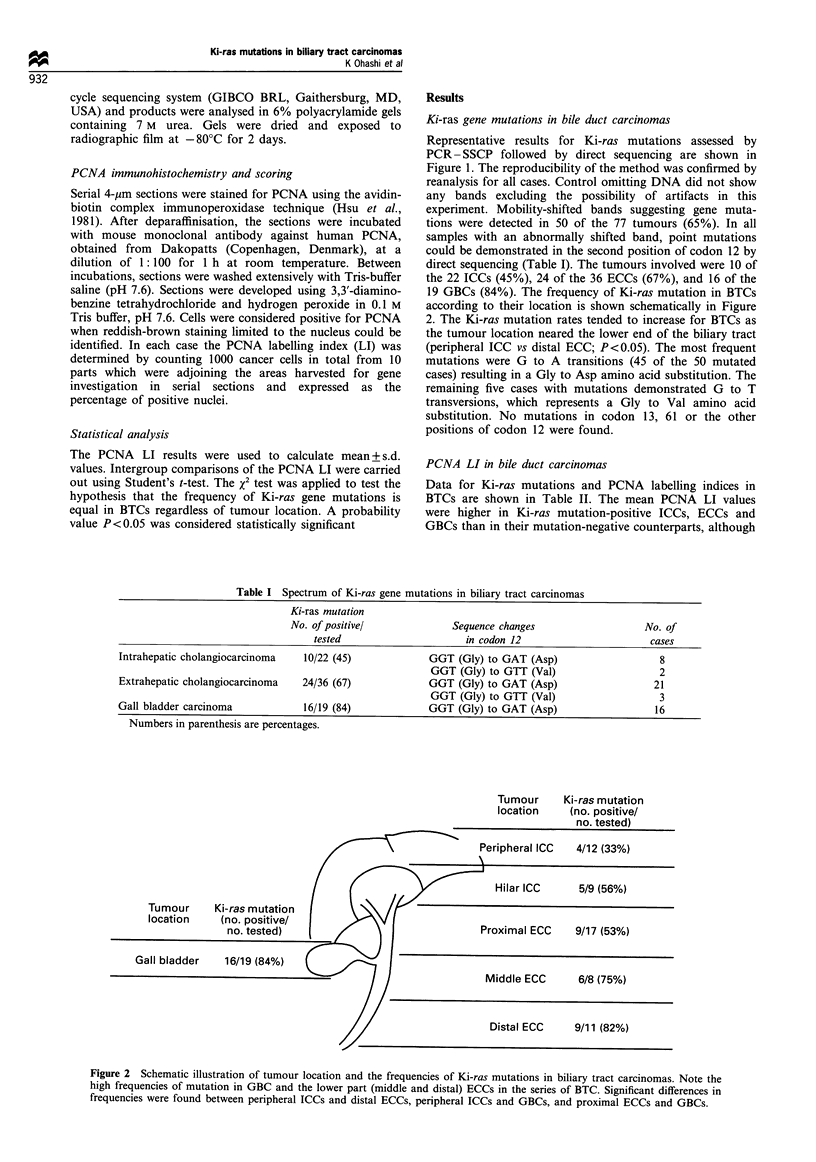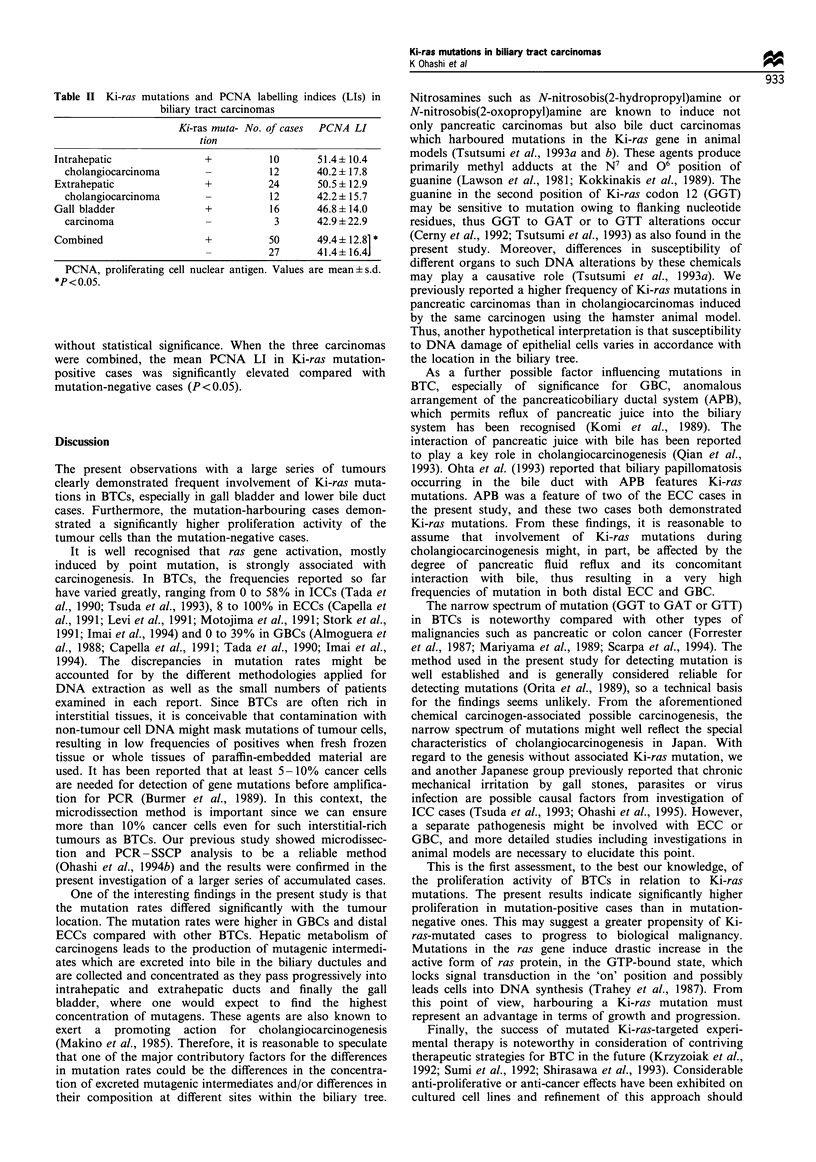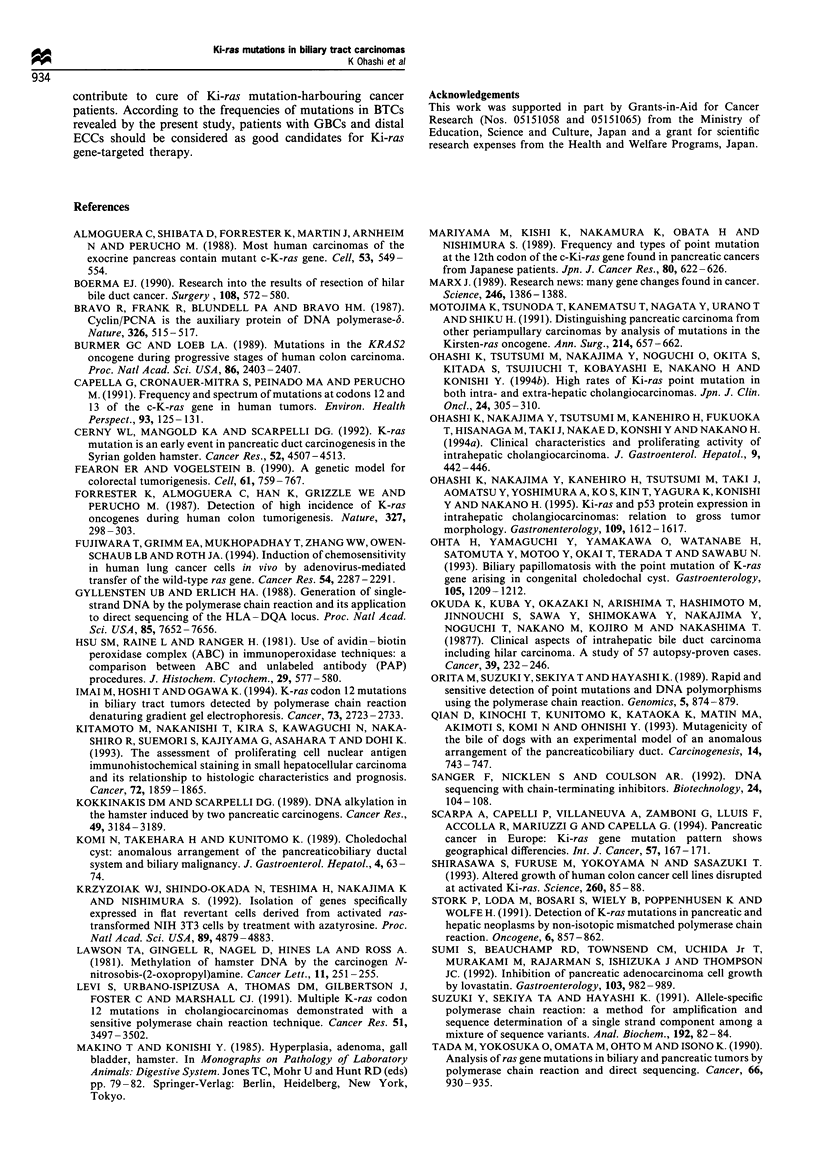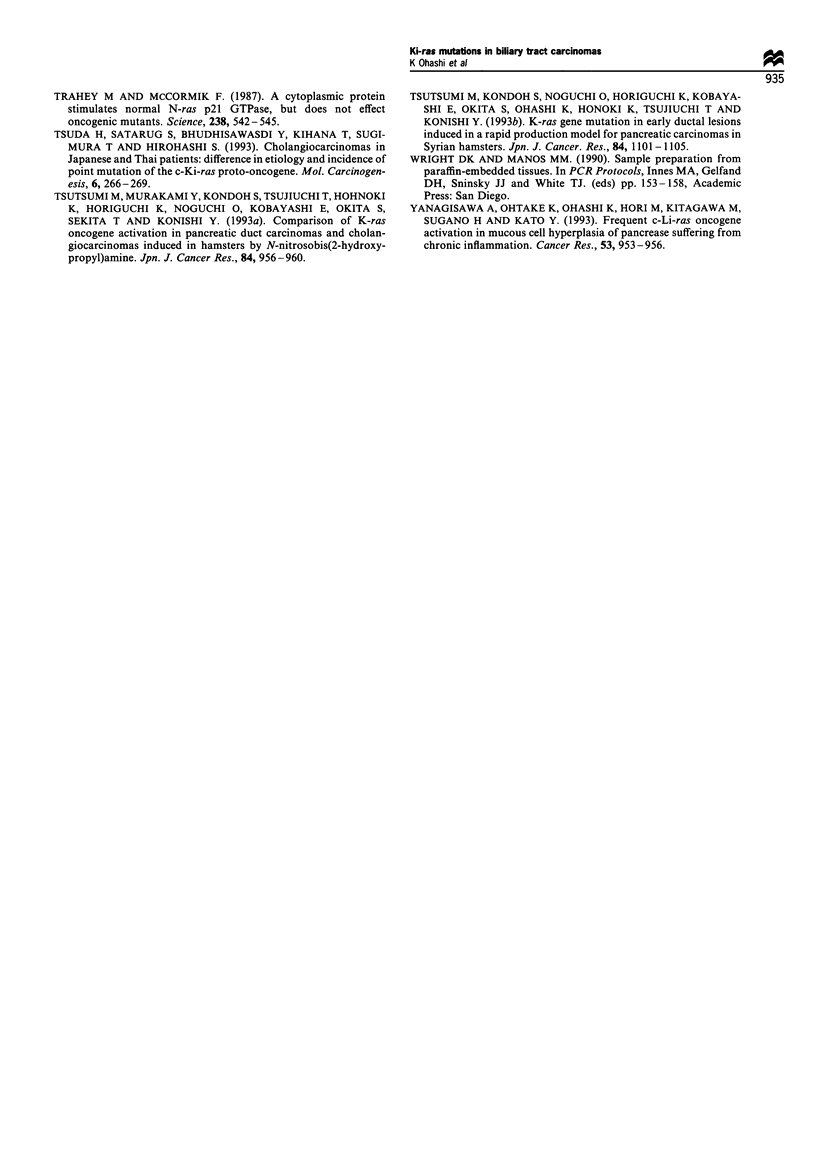# Ki-ras point mutations and proliferation activity in biliary tract carcinomas.

**DOI:** 10.1038/bjc.1996.459

**Published:** 1996-09

**Authors:** K. Ohashi, M. Tstsumi, Y. Nakajima, H. Nakano, Y. Konishi

**Affiliations:** First Department of Surgery, Nara Medical University, Japan.

## Abstract

**Images:**


British Journal of Cancer (1996) 74, 930-935
© 1996 Stockton Press All rights reserved 0007-0920/96 $12.00

### Ki-*ras* point mutations and proliferation activity in biliary tract carcinomas

K Ohashi[1,2], M Tsutsumi[2], Y Nakajima[1], H Nakano[1] and Y Konishi[2]

[1]*First Department of Surgery and* [2]*Department of Oncological Pathology, Nara Medical University, Shijo-cho, Kashihara, Nara 634, Japan.*

**Summary** The association between Ki-*ras* mutations and proliferation activity was investigated in a comprehensive series of biliary tract carcinomas (BTCs). We precisely microdissected samples of tissue from paraffin-embedded sections of 77 BTCs including 22 intrahepatic cholangiocarcinomas (ICCs), 36 extrahepatic cholangiocarcinomas (ECCs), and 19 gall bladder carcinomas (GBCs). Ki-*ras* mutations at exons 1 and 2 were determined by the polymerase chain reaction–single strand conformation polymorphism (PCR–SSCP) method and confirmed by direct sequencing. Proliferation activity was immunohistochemically assessed to generate proliferating cell nuclear antigen labelling indices (PCNA LIs). Ki-*ras* mutations were detected in 10 of 22 ICCs (45%), 24 of 36 ECCs (67%), and in 16 of 19 GBCs (84%). The frequency of Ki-*ras* mutations in peripheral type ICCs was 33% (4 of 12) and that in the hilar type ICCs was 60% (6 of 10). In ECCs the highest value of 82% (9 of 11) was found for carcinomas located in the lower part of the biliary tree. Mean PCNA LI in mutation-positive BTCs was significantly elevated compared with the mutation-negative value. These results indicate frequent involvement of Ki-*ras* mutations in BTCs, especially in GBCs and in distal ECCs, and that carcinomas harbouring a mutation feature high cell proliferation activity.

**Keywords:** biliary tract carcinoma; Ki-*ras* gene; proliferating cell nuclear antigen; cholangiocarcinogenesis; polymerase chain reaction–single strand conformation polymorphism

In the last decade, the significance of alterations of oncogenes or tumour-suppressor genes has become a paradigm in cancer research. It is now generally accepted that accumulation of damage to critical regulatory genes in a multistep manner is the essential mechanism of human tumour pathogenesis (Marx, 1989; Fearon *et al.*, 1990). Recent investigations of suppressor gene transfection or blocking the function of mutated oncogenes have provided strong support for this conclusion and offer clues to new anti-cancer treatment approaches (Krzyzoiak *et al.*, 1992; Sumi *et al.*, 1992; Shirasawa *et al.*, 1993; Fujiwara *et al.*, 1994). For example, abrogating the mutated-*ras*-mediated pathway which exerts transforming activity has been reported to be effective for growth inhibition, at least in *in vitro* models.

Biliary tract carcinomas (BTCs) are characterised by a poor prognosis, because of difficulties in early detection and radical surgical removal as well as being refractory to chemo- and radiotherapy (Boerma, 1990; Ohashi *et al.*, 1994*a*). Basic information on gene alterations is therefore required to understand the mechanisms of their pathogenesis and to facilitate development of genetic approaches to therapy. In terms of gene target therapy, mutated-*ras* has been considered as a candidate from several reports of experimental therapy, describing disruption of the Ki-*ras* gene or depletion of farnesyl isoprenoid to inhibit mutated-*ras*-mediated cell growth (Krzyzoiak *et al.*, 1992; Sumi *et al.*, 1992; Shirasawa *et al.*, 1993). Thus, an accurate estimation of the frequency of association between Ki-*ras* gene mutations and BTCs is important.

The reported frequencies of Ki-*ras* mutations in BTC have varied greatly (Almoguera *et al.*, 1988; Tada *et al.*, 1990; Capella *et al.*, 1991; Levi *et al.*, 1991; Motojima *et al.*, 1991; Stork *et al.*, 1991; Tsuda *et al.*, 1993; Imai *et al.*, 1994) and the discrepancies require explanation. In most cases, whole materials from sectioned formalin-fixed paraffin-embedded specimens of fresh frozen tissues were used for DNA extraction. The possibility of contamination with quantities of DNA from the interstitial tissue which is prominent in BTCs must be taken into account. We previously reported consistently high frequencies of Ki-*ras* gene mutations in both intrahepatic cholangiocarcinomas (ICCs) and extrahepatic cholangiocarcinomas (ECCs) using DNA samples extracted from microdissected tissues under the light microscope (Ohashi *et al.*, 1994*b*). This approach allows the nature of the sampled cell population to be assessed more accurately.

In the present investigation, we extended our previous studies and estimated the mutation rates of a large series of accumulated BTC cases including gall bladder carcinomas (GBCs). In addition, the study included an evaluation of the possible correlation between Ki-*ras* mutation and proliferation activity of tumour cells as determined by proliferating cell nuclear antigen (PCNA) staining, since an understanding of proliferation activity of the tumour cells is very important to provide evidence of biological malignancy (Kitamoto *et al.*, 1993; Ohashi *et al.*, 1994*a*). PCNA, a nuclear auxiliary protein for DNA polymerase-$\delta$ which is closely linked to the cell cycle, is now generally recognised as a useful marker for detecting proliferating cells (Bravo *et al.*, 1987).

### Materials and methods

### *Tumour materials*

Formalin-fixed, paraffin-embedded tissue material from 77 BTC patients in the pathological archives of Nara Medical University and its satellite hospitals was used for the analysis. The tumours comprised 22 ICCs, 36 ECCs and 19 GBCs. The specimens were obtained at surgical treatment between 1982 and 1994. In all cases the location and origin of the tumours could be confirmed by clinical and pathological examinations and pancreatic or papilla Vater carcinomas were not included. ICCs were divided into two groups and ECCs into three groups depending on the location of the tumour origin. According to the classification of Okuda *et al.* (1977), a peripheral ICC ($n=12$) is defined as a tumour originating from the bile duct peripheral to the second fork and a hilar ICC ($n=10$) is one originating from the bile duct between the second fork and the hepatic hilus. Proximal ($n=17$), middle ($n=8$) and distal ($n=11$) ECCs are defined as tumours originating from the bile duct between the hepatic hilus and the cystic duct junction, the cystic duct junction

Correspondence: Y Konoshi, Department of Oncological Pathology, Nara Medical University, 840 Shijo-cho, Kashihara, Nara 634, Japan
Received 21 September 1995; revised 15 March 1996; accepted 2 April 1996

and the upper margin of the pancreas, and the upper margin of the pancreas and the ampullar region respectively. To eliminate bias in interpretation of gene alterations, the divisions of ICCs and ECCs were performed by surgeons and pathologists without knowledge of the results of the gene alterations by unanimous consent.

### *DNA extraction and amplification*

A microdissection method was used for tissue harvesting as previously described (Tsutsumi *et al.*, 1993*b*; Yanagisawa *et al.*, 1993; Ohashi *et al.*, 1994). Briefly, serial sections 4- and 20-$\mu$m thick were cut and attached to slide glasses. The 4 $\mu$m sections were stained with haematoxylin and eosin (H&E) to confirm the presence of carcinoma tissues. The 20-$\mu$m sections were stained with haematoxylin after deparaffinisation. With comparative microscopic observation of the H&E stained section for orientation, tumour cells were cut out using 23-G syringe needles, excluding mesenchymal cells as far as possible. As a countermeasure against heterogeneity within single tumours, ten parts in each case were arbitrarily selected from peripheral and central regions and approximately 1000 cells per case were harvested. These samples were cleaned with ethanol, completely dried and incubated in 400 ml of lysis buffer [10 mM Tris-HCl (pH 8.3), 50 mM potassium chloride, 2.5 mM magnesium chloride, 0.45% Tween 20) containing proteinase K (0.5 mg ml$^{-1}$) for 48 h at 55°C following the protocol described by Wright and Manos (1990). A 159-bp fragment of the first exon of the Ki-*ras* gene was amplified by polymerase chain reaction (PCR) using the primers: GGAATTCGACTGAATATAAACTTG TGG and GGAATTCCTGCACCAGTAATATGC. To target codon 61 of Ki-*ras*, which belongs to exon 2, the following primers yielding a 116 base pair were used: GGAATTCCTACAGGAAGCAAGTAG and GGAATTC-CTCATGTACTGGTCCCT. After denaturation for 3 min at 95°C 500 ng DNAs or distilled water without DNA as an internal control were allowed to run for 60 s at 92°C, 120 s at 55°C and 120 s at 72°C for 40 cycles.

### *SSCP and sequencing analysis*

The PCR products of 159 and 116 bp were analysed by single strand conformation polymorphism (SSCP). Five per cent polyacrylamide gels containing 45 mM Tris-borate (pH 8.3), 4 mM EDTA, and 10% glycerol were prepared, and gel electrophoresis performed at 30W for about 4 h both at 5°C and 30°C constant (LKB Macrophore DNA electrophoresis system, Pharmacia Co, Ltd., Sweden). Gels were dried and exposed to radiographic film at −80°C for about 2 days.

To determine nucleotide sequence alterations detected by mobility shifts in PCR−SSCP analysis, we sequenced the DNAs after their amplification by asymmetric PCR (Gyllensten *et al.*, 1988). DNA fragments showing a mobility shift compared with the normal control by PCR−SSCP analysis were separated and eluted from the polyacrylamide gel according to the method of Suzuki *et al.* (1991). After subsequent PCR using the same primers as described above, amplification reaction mixtures were purified with a Microcon (Amicon, Denvers, MA, USA). For determination of nucleotide sequences, the dideoxy chain-termination method (Sanger *et al.*, 1992) was performed using a dsDNA

**Figure 1** PCR−SSCP analysis of the Ki-*ras* gene in biliary tract carcinomas (exon 1). (**a**) Representative SSCP results of 22 cases. Fragments with abnormal mobility shifts are evident in cases 1, 3, 4, 6, 7, 9, 11, 13, 15, 18, 19, 21 and 22 (arrowheads). IC, internal control without adding DNA. (**b**) Representative direct sequencing results around codon 12 of the Ki-*ras* gene of control, cases 1, 3, 4 and 9. Cases 1 and 4 demonstrate G to A transitions in the second position (arrowhead). Cases 4 and a G to T transversion in the second position (arrowhead). The sequencing results of cases 6, 7, 11, 13, 19, 21 and 22 were the same as for cases 1 and 4 and the results of cases 9, 15 and 18 were the same as for case 3 (figures not shown).

cycle sequencing system (GIBCO BRL, Gaithersburg, MD, USA) and products were analysed in 6% polyacrylamide gels containing 7 M urea. Gels were dried and exposed to radiographic film at −80°C for 2 days.

*PCNA immunohistochemistry and scoring*

Serial 4-μm sections were stained for PCNA using the avidin-biotin complex immunoperoxidase technique (Hsu *et al.*, 1981). After deparaffinisation, the sections were incubated with mouse monoclonal antibody against human PCNA, obtained from Dakopatts (Copenhagen, Denmark), at a dilution of 1:100 for 1 h at room temperature. Between incubations, sections were washed extensively with Tris-buffer saline (pH 7.6). Sections were developed using 3,3′-diamino-benzine tetrahydrochloride and hydrogen peroxide in 0.1 M Tris buffer, pH 7.6. Cells were considered positive for PCNA when reddish-brown staining limited to the nucleus could be identified. In each case the PCNA labelling index (LI) was determined by counting 1000 cancer cells in total from 10 parts which were adjoining the areas harvested for gene investigation in serial sections and expressed as the percentage of positive nuclei.

*Statistical analysis*

The PCNA LI results were used to calculate mean±s.d. values. Intergroup comparisons of the PCNA LI were carried out using Student's *t*-test. The $\chi^2$ test was applied to test the hypothesis that the frequency of Ki-*ras* gene mutations is equal in BTCs regardless of tumour location. A probability value $P<0.05$ was considered statistically significant

### Results

*Ki-*ras *gene mutations in bile duct carcinomas*

Representative results for Ki-*ras* mutations assessed by PCR–SSCP followed by direct sequencing are shown in Figure 1. The reproducibility of the method was confirmed by reanalysis for all cases. Control omitting DNA did not show any bands excluding the possibility of artifacts in this experiment. Mobility-shifted bands suggesting gene mutations were detected in 50 of the 77 tumours (65%). In all samples with an abnormally shifted band, point mutations could be demonstrated in the second position of codon 12 by direct sequencing (Table I). The tumours involved were 10 of the 22 ICCs (45%), 24 of the 36 ECCs (67%), and 16 of the 19 GBCs (84%). The frequency of Ki-*ras* mutation in BTCs according to their location is shown schematically in Figure 2. The Ki-*ras* mutation rates tended to increase for BTCs as the tumour location neared the lower end of the biliary tract (peripheral ICC *vs* distal ECC; $P<0.05$). The most frequent mutations were G to A transitions (45 of the 50 mutated cases) resulting in a Gly to Asp amino acid substitution. The remaining five cases with mutations demonstrated G to T transversions, which represents a Gly to Val amino acid substitution. No mutations in codon 13, 61 or the other positions of codon 12 were found.

*PCNA LI in bile duct carcinomas*

Data for Ki-*ras* mutations and PCNA labelling indices in BTCs are shown in Table II. The mean PCNA LI values were higher in Ki-*ras* mutation-positive ICCs, ECCs and GBCs than in their mutation-negative counterparts, although

**Table I** Spectrum of Ki-*ras* gene mutations in biliary tract carcinomas

| | *Ki-*ras *mutation No. of positive/ tested* | *Sequence changes in codon 12* | *No. of cases* |
|---|---|---|---|
| Intrahepatic cholangiocarcinoma | 10/22 (45) | GGT (Gly) to GAT (Asp) | 8 |
| | | GGT (Gly) to GTT (Val) | 2 |
| Extrahepatic cholangiocarcinoma | 24/36 (67) | GGT (Gly) to GAT (Asp) | 21 |
| | | GGT (Gly) to GTT (Val) | 3 |
| Gall bladder carcinoma | 16/19 (84) | GGT (Gly) to GAT (Asp) | 16 |

Numbers in parenthesis are percentages.

**Figure 2** Schematic illustration of tumour location and the frequencies of Ki-*ras* mutations in biliary tract carcinomas. Note the high frequencies of mutation in GBC and the lower part (middle and distal) ECCs in the series of BTC. Significant differences in frequencies were found between peripheral ICCs and distal ECCs, peripheral ICCs and GBCs, and proximal ECCs and GBCs.

**Table II** Ki-*ras* mutations and PCNA labelling indices (LIs) in biliary tract carcinomas

| | *Ki-*ras *mutation* | *No. of cases* | *PCNA LI* |
|---|---|---|---|
| Intrahepatic | + | 10 | 51.4±10.4 |
| cholangiocarcinoma | − | 12 | 40.2±17.8 |
| Extrahepatic | + | 24 | 50.5±12.9 |
| cholangiocarcinoma | − | 12 | 42.2±15.7 |
| Gall bladder | + | 16 | 46.8±14.0 |
| carcinoma | − | 3 | 42.9±22.9 |
| Combined | + | 50 | 49.4±12.8 * |
| | − | 27 | 41.4±16.4 |

PCNA, proliferating cell nuclear antigen. Values are mean±s.d. *$P<0.05$.

without statistical significance. When the three carcinomas were combined, the mean PCNA LI in Ki-*ras* mutation-positive cases was significantly elevated compared with mutation-negative cases ($P<0.05$).

### Discussion

The present observations with a large series of tumours clearly demonstrated frequent involvement of Ki-*ras* mutations in BTCs, especially in gall bladder and lower bile duct cases. Furthermore, the mutation-harbouring cases demonstrated a significantly higher proliferation activity of the tumour cells than the mutation-negative cases.

It is well recognised that *ras* gene activation, mostly induced by point mutation, is strongly associated with carcinogenesis. In BTCs, the frequencies reported so far have varied greatly, ranging from 0 to 58% in ICCs (Tada *et al*., 1990; Tsuda *et al*., 1993), 8 to 100% in ECCs (Capella *et al*., 1991; Levi *et al*., 1991; Motojima *et al*., 1991; Stork *et al*., 1991; Imai *et al*., 1994) and 0 to 39% in GBCs (Almoguera *et al*., 1988; Capella *et al*., 1991; Tada *et al*., 1990; Imai *et al*., 1994). The discrepancies in mutation rates might be accounted for by the different methodologies applied for DNA extraction as well as the small numbers of patients examined in each report. Since BTCs are often rich in interstitial tissues, it is conceivable that contamination with non-tumour cell DNA might mask mutations of tumour cells, resulting in low frequencies of positives when fresh frozen tissue or whole tissues of paraffin-embedded material are used. It has been reported that at least 5−10% cancer cells are needed for detection of gene mutations before amplification for PCR (Burmer *et al*., 1989). In this context, the microdissection method is important since we can ensure more than 10% cancer cells even for such interstitial-rich tumours as BTCs. Our previous study showed microdissection and PCR−SSCP analysis to be a reliable method (Ohashi *et al*., 1994*b*) and the results were confirmed in the present investigation of a larger series of accumulated cases.

One of the interesting findings in the present study is that the mutation rates differed significantly with the tumour location. The mutation rates were higher in GBCs and distal ECCs compared with other BTCs. Hepatic metabolism of carcinogens leads to the production of mutagenic intermediates which are excreted into bile in the biliary ductules and are collected and concentrated as they pass progressively into intrahepatic and extrahepatic ducts and finally the gall bladder, where one would expect to find the highest concentration of mutagens. These agents are also known to exert a promoting action for cholangiocarcinogenesis (Makino *et al*., 1985). Therefore, it is reasonable to speculate that one of the major contributory factors for the differences in mutation rates could be the differences in the concentration of excreted mutagenic intermediates and/or differences in their composition at different sites within the biliary tree. Nitrosamines such as *N*-nitrosobis(2-hydropropyl)amine or *N*-nitrosobis(2-oxopropyl)amine are known to induce not only pancreatic carcinomas but also bile duct carcinomas which harboured mutations in the Ki-*ras* gene in animal models (Tsutsumi *et al*., 1993*a* and *b*). These agents produce primarily methyl adducts at the $N^7$ and $O^6$ position of guanine (Lawson *et al*., 1981; Kokkinakis *et al*., 1989). The guanine in the second position of Ki-*ras* codon 12 (GGT) may be sensitive to mutation owing to flanking nucleotide residues, thus GGT to GAT or to GTT alterations occur (Cerny *et al*., 1992; Tsutsumi *et al*., 1993) as also found in the present study. Moreover, differences in susceptibility of different organs to such DNA alterations by these chemicals may play a causative role (Tsutsumi *et al*., 1993*a*). We previously reported a higher frequency of Ki-*ras* mutations in pancreatic carcinomas than in cholangiocarcinomas induced by the same carcinogen using the hamster animal model. Thus, another hypothetical interpretation is that susceptibility to DNA damage of epithelial cells varies in accordance with the location in the biliary tree.

As a further possible factor influencing mutations in BTC, especially of significance for GBC, anomalous arrangement of the pancreaticobiliary ductal system (APB), which permits reflux of pancreatic juice into the biliary system has been recognised (Komi *et al*., 1989). The interaction of pancreatic juice with bile has been reported to play a key role in cholangiocarcinogenesis (Qian *et al*., 1993). Ohta *et al*. (1993) reported that biliary papillomatosis occurring in the bile duct with APB features Ki-*ras* mutations. APB was a feature of two of the ECC cases in the present study, and these two cases both demonstrated Ki-*ras* mutations. From these findings, it is reasonable to assume that involvement of Ki-*ras* mutations during cholangiocarcinogenesis might, in part, be affected by the degree of pancreatic fluid reflux and its concomitant interaction with bile, thus resulting in a very high frequencies of mutation in both distal ECC and GBC.

The narrow spectrum of mutation (GGT to GAT or GTT) in BTCs is noteworthy compared with other types of malignancies such as pancreatic or colon cancer (Forrester *et al*., 1987; Mariyama *et al*., 1989; Scarpa *et al*., 1994). The method used in the present study for detecting mutation is well established and is generally considered reliable for detecting mutations (Orita *et al*., 1989), so a technical basis for the findings seems unlikely. From the aforementioned chemical carcinogen-associated possible carcinogenesis, the narrow spectrum of mutations might well reflect the special characteristics of cholangiocarcinogenesis in Japan. With regard to the genesis without associated Ki-*ras* mutation, we and another Japanese group previously reported that chronic mechanical irritation by gall stones, parasites or virus infection are possible causal factors from investigation of ICC cases (Tsuda *et al*., 1993; Ohashi *et al*., 1995). However, a separate pathogenesis might be involved with ECC or GBC, and more detailed studies including investigations in animal models are necessary to elucidate this point.

This is the first assessment, to the best our knowledge, of the proliferation activity of BTCs in relation to Ki-*ras* mutations. The present results indicate significantly higher proliferation in mutation-positive cases than in mutation-negative ones. This may suggest a greater propensity of Ki-*ras*-mutated cases to progress to biological malignancy. Mutations in the *ras* gene induce drastic increase in the active form of *ras* protein, in the GTP-bound state, which locks signal transduction in the 'on' position and possibly leads cells into DNA synthesis (Trahey *et al*., 1987). From this point of view, harbouring a Ki-*ras* mutation must represent an advantage in terms of growth and progression.

Finally, the success of mutated Ki-*ras*-targeted experimental therapy is noteworthy in consideration of contriving therapeutic strategies for BTC in the future (Krzyzoiak *et al*., 1992; Sumi *et al*., 1992; Shirasawa *et al*., 1993). Considerable anti-proliferative or anti-cancer effects have been exhibited on cultured cell lines and refinement of this approach should contribute to cure of Ki-*ras* mutation-harbouring cancer patients. According to the frequencies of mutations in BTCs revealed by the present study, patients with GBCs and distal ECCs should be considered as good candidates for Ki-*ras* gene-targeted therapy.

**Acknowledgements**
This work was supported in part by Grants-in-Aid for Cancer Research (Nos. 05151058 and 05151065) from the Ministry of Education, Science and Culture, Japan and a grant for scientific research expenses from the Health and Welfare Programs, Japan.

### References

ALMOGUERA C, SHIBATA D, FORRESTER K, MARTIN J, ARNHEIM N AND PERUCHO M. (1988). Most human carcinomas of the exocrine pancreas contain mutant c-K-*ras* gene. *Cell*, **53**, 549-554.

BOERMA EJ. (1990). Research into the results of resection of hilar bile duct cancer. *Surgery* , **108**, 572-580.

BRAVO R, FRANK R, BLUNDELL PA AND BRAVO HM. (1987). Cyclin/PCNA is the auxiliary protein of DNA polymerase-$\delta$. *Nature*, **326**, 515-517.

BURMER GC AND LOEB LA. (1989). Mutations in the *KRAS2* oncogene during progressive stages of human colon carcinoma. *Proc. Natl Acad. Sci. USA*, **86**, 2403-2407.

CAPELLA G, CRONAUER-MITRA S, PEINADO MA AND PERUCHO M. (1991). Frequency and spectrum of mutations at codons 12 and 13 of the c-K-*ras* gene in human tumors. *Environ. Health Perspect.*, **93**, 125-131.

CERNY WL, MANGOLD KA AND SCARPELLI DG. (1992). K-*ras* mutation is an early event in pancreatic duct carcinogenesis in the Syrian golden hamster. *Cancer Res.*, **52**, 4507-4513.

FEARON ER AND VOGELSTEIN B. (1990). A genetic model for colorectal tumorigenesis. *Cell*, **61**, 759-767.

FORRESTER K, ALMOGUERA C, HAN K, GRIZZLE WE AND PERUCHO M. (1987). Detection of high incidence of K-*ras* oncogenes during human colon tumorigenesis. *Nature*, **327**, 298-303.

FUJIWARA T, GRIMM EA, MUKHOPADHAY T, ZHANG WW, OWEN-SCHAUB LB AND ROTH JA. (1994). Induction of chemosensitivity in human lung cancer cells *in vivo* by adenovirus-mediated transfer of the wild-type *ras* gene. *Cancer Res.* **54**, 2287-2291.

GYLLENSTEN UB AND ERLICH HA. (1988). Generation of single-strand DNA by the polymerase chain reaction and its application to direct sequencing of the HLA-DQA locus. *Proc. Natl Acad. Sci. USA*, **85**, 7652-7656.

HSU SM, RAINE L AND RANGER H. (1981). Use of avidin-biotin peroxidase complex (ABC) in immunoperoxidase techniques: a comparison between ABC and unlabeled antibody (PAP) procedures. *J. Histochem. Cytochem.*, **29**, 577-580.

IMAI M, HOSHI T AND OGAWA K. (1994). K-*ras* codon 12 mutations in biliary tract tumors detected by polymerase chain reaction denaturing gradient gel electrophoresis. *Cancer*, **73**, 2723-2733.

KITAMOTO M, NAKANISHI T, KIRA S, KAWAGUCHI N, NAKA-SHIRO R, SUEMORI S, KAJIYAMA G, ASAHARA T AND DOHI K. (1993). The assessment of proliferating cell nuclear antigen immunohistochemical staining in small hepatocellular carcinoma and its relationship to histologic characteristics and prognosis. *Cancer*, **72**, 1859-1865.

KOKKINAKIS DM AND SCARPELLI DG. (1989). DNA alkylation in the hamster induced by two pancreatic carcinogens. *Cancer Res.*, **49**, 3184-3189.

KOMI N, TAKEHARA H AND KUNITOMO K. (1989). Choledochal cyst: anomalous arrangement of the pancreaticobiliary ductal system and biliary malignancy. *J. Gastroenterol. Hepatol.*, **4**, 63-74.

KRZYZOIAK WJ, SHINDO-OKADA N, TESHIMA H, NAKAJIMA K AND NISHIMURA S. (1992). Isolation of genes specifically expressed in flat revertant cells derived from activated *ras*-transformed NIH 3T3 cells by treatment with azatyrosine. *Proc. Natl Acad. Sci. USA*, **89**, 4879-4883.

LAWSON TA, GINGELL R, NAGEL D, HINES LA AND ROSS A. (1981). Methylation of hamster DNA by the carcinogen *N*-nitrosobis-(2-oxopropyl)amine. *Cancer Lett.*, **11**, 251-255.

LEVI S, URBANO-ISPIZUSA A, THOMAS DM, GILBERTSON J, FOSTER C AND MARSHALL CJ. (1991). Multiple K-*ras* codon 12 mutations in cholangiocarcinomas demonstrated with a sensitive polymerase chain reaction technique. *Cancer Res.* **51**, 3497-3502.

MAKINO T AND KONISHI Y. (1985). Hyperplasia, adenoma, gall bladder, hamster. In *Monographs on Pathology of Laboratory Animals: Digestive System.* Jones TC, Mohr U and Hunt RD (eds) pp. 79-82. Springer-Verlag: Berlin, Heidelberg, New York, Tokyo.

MARIYAMA M, KISHI K, NAKAMURA K, OBATA H AND NISHIMURA S. (1989). Frequency and types of point mutation at the 12th codon of the c-Ki-*ras* gene found in pancreatic cancers from Japanese patients. *Jpn. J. Cancer Res.*, **80**, 622-626.

MARX J. (1989). Research news: many gene changes found in cancer. *Science*, **246**, 1386-1388.

MOTOJIMA K, TSUNODA T, KANEMATSU T, NAGATA Y, URANO T AND SHIKU H. (1991). Distinguishing pancreatic carcinoma from other periampullary carcinomas by analysis of mutations in the Kirsten-*ras* oncogene. *Ann. Surg.*, **214**, 657-662.

OHASHI K, TSUTSUMI M, NAKAJIMA Y, NOGUCHI O, OKITA S, KITADA S, TSUJIUCHI T, KOBAYASHI E, NAKANO H AND KONISHI Y. (1994*b*). High rates of Ki-*ras* point mutation in both intra- and extra-hepatic cholangiocarcinomas. *Jpn. J. Clin. Oncl.*, **24**, 305-310.

OHASHI K, NAKAJIMA Y, TSUTSUMI M, KANEHIRO H, FUKUOKA T, HISANAGA M, TAKI J, NAKAE D, KONSHI Y AND NAKANO H. (1994*a*). Clinical characteristics and proliferating activity of intrahepatic cholangiocarcinoma. *J. Gastroenterol. Hepatol.*, **9**, 442-446.

OHASHI K, NAKAJIMA Y, KANEHIRO H, TSUTSUMI M, TAKI J, AOMATSU Y, YOSHIMURA A, KO S, KIN T, YAGURA K, KONISHI Y AND NAKANO H. (1995). Ki-*ras* and p53 protein expression in intrahepatic cholangiocarcinomas: relation to gross tumor morphology. *Gastronenterology*, **109**, 1612-1617.

OHTA H, YAMAGUCHI Y, YAMAKAWA O, WATANABE H, SATOMUTA Y, MOTOO Y, OKAI T, TERADA T AND SAWABU N. (1993). Biliary papillomatosis with the point mutation of K-*ras* gene arising in congenital choledochal cyst. *Gastroenterology*, **105**, 1209-1212.

OKUDA K, KUBA Y, OKAZAKI N, ARISHIMA T, HASHIMOTO M, JINNOUCHI S, SAWA Y, SHIMOKAWA Y, NAKAJIMA Y, NOGUCHI T, NAKANO M, KOJIRO M AND NAKASHIMA T. (19877). Clinical aspects of intrahepatic bile duct carcinoma including hilar carcinoma. A study of 57 autopsy-proven cases. *Cancer*, **39**, 232-246.

ORITA M, SUZUKI Y, SEKIYA T AND HAYASHI K. (1989). Rapid and sensitive detection of point mutations and DNA polymorphisms using the polymerase chain reaction. *Genomics*, **5**, 874-879.

QIAN D, KINOCHI T, KUNITOMO K, KATAOKA K, MATIN MA, AKIMOTI S, KOMI N AND OHNISHI Y. (1993). Mutagenicity of the bile of dogs with an experimental model of an anomalous arrangement of the pancreaticobiliary duct. *Carcinogenesis*, **14**, 743-747.

SANGER F, NICKLEN S AND COULSON AR. (1992). DNA sequencing with chain-terminating inhibitors. *Biotechnology*, **24**, 104-108.

SCARPA A, CAPELLI P, VILLANEUVA A, ZAMBONI G, LLUIS F, ACCOLLA R, MARIUZZI G AND CAPELLA G. (1994). Pancreatic cancer in Europe: Ki-*ras* gene mutation pattern shows geographical differencies. *Int. J. Cancer*, **57**, 167-171.

SHIRASAWA S, FURUSE M, YOKOYAMA N AND SASAZUKI T. (1993). Altered growth of human colon cancer cell lines disrupted at activated Ki-*ras*. *Science*, **260**, 85-88.

STORK P, LODA M, BOSARI S, WIELY B, POPPENHUSEN K AND WOLFE H. (1991). Detection of K-*ras* mutations in pancreatic and hepatic neoplasms by non-isotopic mismatched polymerase chain reaction. *Oncogene*, **6**, 857-862.

SUMI S, BEAUCHAMP RD, TOWNSEND CM, UCHIDA Jr T, MURAKAMI M, RAJARMAN S, ISHIZUKA J AND THOMPSON JC. (1992). Inhibition of pancreatic adenocarcinoma cell growth by lovastatin. *Gastroenterology*, **103**, 982-989.

SUZUKI Y, SEKIYA TA AND HAYASHI K. (1991). Allele-specific polymerase chain reaction: a method for amplification and sequence determination of a single strand component among a mixture of sequence variants. *Anal. Biochem.*, **192**, 82-84.

TADA M, YOKOSUKA O, OMATA M, OHTO M AND ISONO K. (1990). Analysis of *ras* gene mutations in biliary and pancreatic tumors by polymerase chain reaction and direct sequencing. *Cancer*, **66**, 930-935.

TRAHEY M AND MCCORMIK F. (1987). A cytoplasmic protein stimulates normal N-*ras* p21 GTPase, but does not effect oncogenic mutants. *Science*, **238**, 542–545.

TSUDA H, SATARUG S, BHUDHISAWASDI Y, KIHANA T, SUGIMURA T AND HIROHASHI S. (1993). Cholangiocarcinomas in Japanese and Thai patients: difference in etiology and incidence of point mutation of the c-Ki-*ras* proto-oncogene. *Mol. Carcinogenesis*, **6**, 266–269.

TSUTSUMI M, MURAKAMI Y, KONDOH S, TSUJIUCHI T, HOHNOKI K, HORIGUCHI K, NOGUCHI O, KOBAYASHI E, OKITA S, SEKITA T AND KONISHI Y. (1993*a*). Comparison of K-*ras* oncogene activation in pancreatic duct carcinomas and cholangiocarcinomas induced in hamsters by *N*-nitrosobis(2-hydroxypropyl)amine. *Jpn. J. Cancer Res.*, **84**, 956–960.

TSUTSUMI M, KONDOH S, NOGUCHI O, HORIGUCHI K, KOBAYASHI E, OKITA S, OHASHI K, HONOKI K, TSUJIUCHI T AND KONISHI Y. (1993*b*). K-*ras* gene mutation in early ductal lesions induced in a rapid production model for pancreatic carcinomas in Syrian hamsters. *Jpn. J. Cancer. Res.*, **84**, 1101–1105.

WRIGHT DK AND MANOS MM. (1990). Sample preparation from paraffin-embedded tissues. In *PCR Protocols*, Innes MA, Gelfand DH, Sninsky JJ and White TJ. (eds) pp. 153–158, Academic Press: San Diego.

YANAGISAWA A, OHTAKE K, OHASHI K, HORI M, KITAGAWA M, SUGANO H AND KATO Y. (1993). Frequent c-Li-*ras* oncogene activation in mucous cell hyperplasia of pancrease suffering from chronic inflammation. *Cancer Res.*, **53**, 953–956.